# Supplementary material for: Reinvestigating the Preferential Enrichment of DL-Arginine Fumarate: New Thoughts on the Mechanism of This Far from Equilibrium Crystallization Phenomenon
Source: Molecules. 2022 Dec 7;27(24):8652. doi: 10.3390/molecules27248652 (PMC9781920; doi:10.3390/molecules27248652)

Reinvestigating the Preferential Enrichment of DL-Arginine Fumarate: New Thoughts on the Mechanism of this far from Equilibrium Crystallization Phenomenon

C. De Saint Jores [1], C. Brandel [2,*], M. Vaccaro [3], N. Gharbi [3], I. Schmitz-Afonso [4], P. Cardinael [3], R. Tamura[5] and G. Coquerel [2,*]

[1] Univ Rouen Normandie, FR3038, SMS , UR 3233, F-76000 Rouen, France. Present adress : Institut de Chimie Organique et Analytique, University of Orléans, CNRS UMR 7311, CEDEX 2, 45067 Orléans, France
[2] Univ Rouen Normandie, SMS , UR 3233, F-76000 Rouen, France
[3] Univ Rouen Normandie, FR3038, SMS , UR 3233, F-76000 Rouen, France
[4] Normandie Univ, COBRA UMR 6014 et FR 3038 Univ Rouen, INSA Rouen, CNRS IRCOF, 1 Rue Tesnière, 76821 Mont-Saint-Aignan Cedex, France
[5] Graduate School of Human and Environmental Studies, Kyoto University, Kyoto 606-8501, Japan
* Correspondence: clement.brandel@univ-rouen.fr; gerard.coquerel@univ-rouen.fr; Tel.: +33235522902

Supporting information

Table S1: Influence of rocking plate

Agitation speed (rpm)	Time (h)	e.e. solid (%)	e.e. liquid (%)
0	144	-2.7	90
0	144	-3.9	94
0	77	-1.3	97
6	144	-1.3	90
6	144	-2	95
6	77	-2.7	97
6	24	-1.5	94
30	144	0	77
30	144	0	77
30	77	-1.2	95
30	24	-1.5	90
30	24	-1.7	97

Table S2: evolution of the system after 6 days

Time	e.e. deposited crystals (%)	e.e. mother liquor (%)
144 h	-2.7	90
144 h	-3.9	94
2 months	-3.0	95

1 year	-1.6	80

Table S3: PE experiments with various supersaturation rates

Supersaturation rate	e.e. solid (%)	e.e. liquid (%)
8	-1.6	93
8	-2.3	90
6	-1.7	89
6	-2.1	90
4	-2	85
4	-2.5	90
2	1.8	66

Table S4: Doping the process with $^{13}C_6$-L-Arginine.HCl, HPLC-MS global results

Addition after 2.5 h D-rich experiment						
Global mass balance	e.e. initial (% D)	m_{Liquid} (mg)	e.e.$_{Liquid}$ (% D)	m_{Solid} (mg)	e.e.$_{Solid}$ (% L)	m_{Total} (mg)
	5.8	56 ±14	91 ±1	547 ±82	3.9 ±0.5	609 ±96
	6.0	56 ±14	91 ±1	668 ±101	2.9 ±0.5	726 ±115
	4.4	56 ±14	91 ±1	623 ±93	2.8 ±0.5	679 ±107
Labeled arginine mass balance	$m_{Initial}$ (µg)	m_{Liquid} (µg)	$\%_{Liquid}$	m_{Solid} (µg)	$\%_{Solid}$	m_{Total} (µg)
	835	34 ±3	5	697 ±146	95	731 ±149
	892	32 ±3	5	647 ±193	95	674 ±196
	837	23 ±3	5	715 ±215	97	739 ±217
Addition after 6 h D-rich experiment						
Global mass balance	e.e. initial (% D)	m_{Liquid} (mg)	e.e.$_{Liquid}$ (% D)	m_{Solid} (mg)	e.e.$_{Solid}$ (% L)	m_{Total} (mg)
	5.4	63 ±15	94 ±1	614 ±93	3.2 ±0.5	677 ±108
	5.6	75 ±18	94 ±1	595 ±89	3.3 ±0.5	670 ±108
Labeled arginine mass balance	$m_{Initial}$ (µg)	m_{Liquid} (µg)	$\%_{Liquid}$	m_{Solid} (µg)	$\%_{Solid}$	m_{Total} (µg)
	839	39 ±4	7	537 ±161	93	576 ±165
	831	34 ±3	6	575 ±173	94	609 ±176
Addition after 6 days D-rich experiment						
Global mass balance	e.e. initial (% D)	m_{Liquid} (mg)	e.e.$_{Liquid}$ (% D)	m_{Solid} (mg)	e.e.$_{Solid}$ (% L)	m_{Total} (mg)
	5.0	68 ±17	94 ±1	575 ±86	4.0 ±0.5	640 ±102
	5.0	66 ±16	94 ±1	631 ±89	3.0 ±0.5	670 ±111
Labeled arginine mass balance	$m_{Initial}$ (µg)	m_{Liquid} (µg)	$\%_{Liquid}$	m_{Solid} (µg)	$\%_{Solid}$	m_{Total} (µg)
	832	185 ±18	26	520 ±156	74	705 ±174
	841	174 ±17	23	600 ±180	77	775 ±197
Addition after 2.5 h L-rich experiment						
Global mass balance	e.e. initial (% L)	m_{Liquid} (mg)	e.e.$_{Liquid}$ (% L)	m_{Solid} (mg)	e.e.$_{Solid}$ (% D)	m_{Total} (mg)
	4.9	68 ±2	80 ±1	743 ±19	3.4 ±0.5	811 ±21
	4.8	71 ±3	96 ±1	739 ±19	3.6 ±0.5	811 ±22
Labeled arginine	$m_{Initial}$ (µg)	m_{Liquid} (µg)	$\%_{Liquid}$	m_{Solid} (µg)	$\%_{Solid}$	m_{Total} (µg)
	827	371 ±37	44	476 ±48	56	847 ±85

mass balance	855	453 ±45	50	447 ±45	50	901 ±90
Addition after 6 days L-rich experiment						
Global mass balance	e.e. initial (% L)	m_{Liquid} (mg)	e.e.$_{Liquid}$ (% L)	m_{Solid} (mg)	e.e.$_{Solid}$ (% D)	m_{Total} (mg)
	4.5	65 ±2	96 ±1	663 ±100	4.4 ±0.5	728 ±103
	4.3	60 ±2	92 ±1	663 ±100	4.0 ±0.5	724 ±102
	4.6	63 ±2	92 ±1	674 ±101	5.0 ±0.5	739 ±103
Labeled arginine mass balance	$m_{Initial}$ (µg)	m_{Liquid} (µg)	$\%_{Liquid}$	m_{Solid} (µg)	$\%_{Solid}$	m_{Total} (µg)
	898	572 ±40	84	110 ±33	16	682 ±77
	883	791 ±79	93	61 ±18	7	852 ±97
	824	567 ±57	81	130 ±39	19	698 ±96

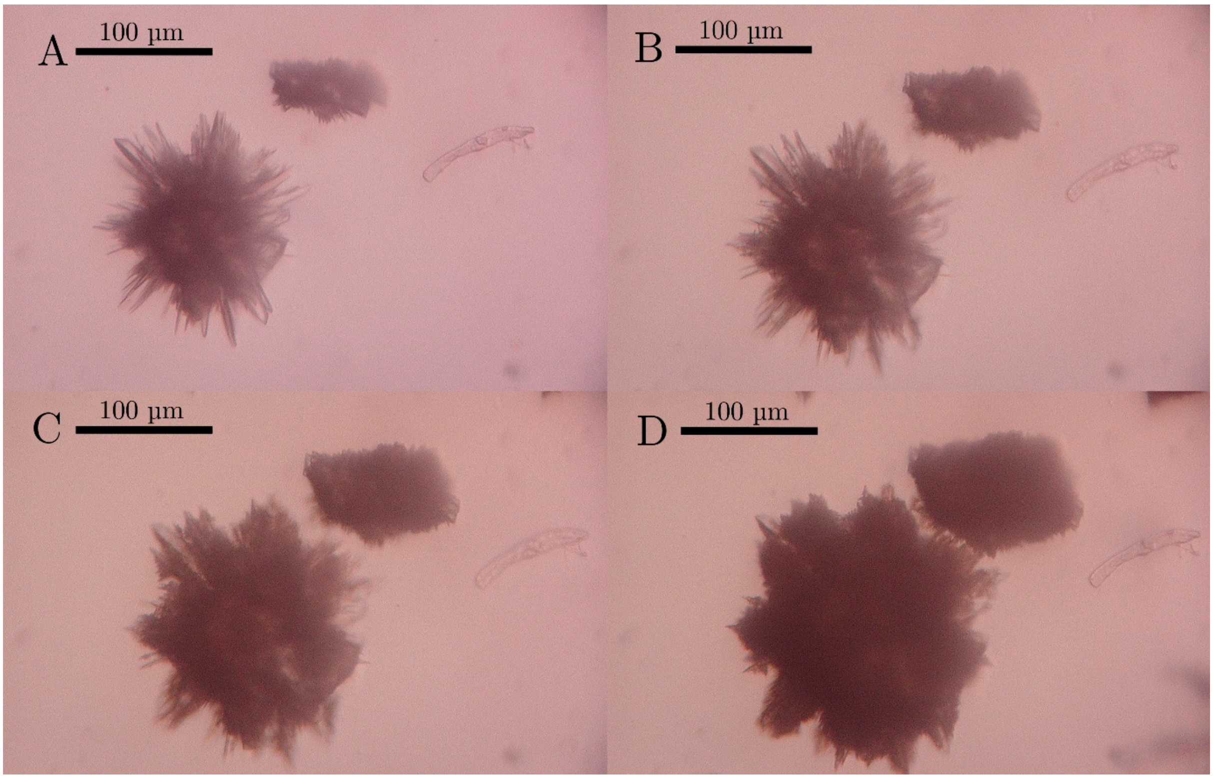

Figure S1: Monitoring by microscopy (Hirox HK7700) Growth of ArgFum salts at 5°Cafter 1 min, 1.5 min, 1.8 min and 2.7 min in PE conditions

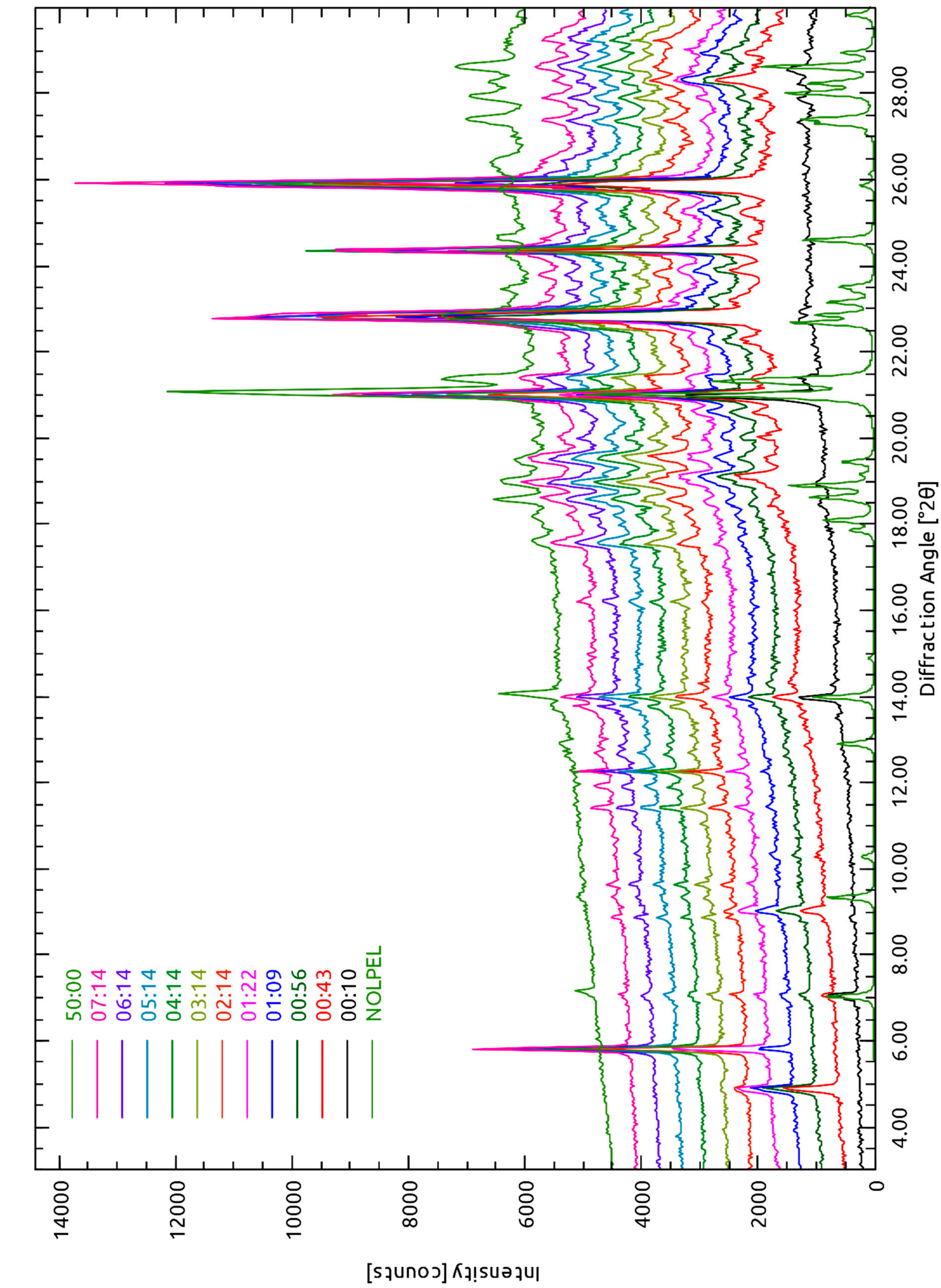

Figure S2: In-SituX XRPD patterns, e.e.$_{solid}$=2.0% D, e.e.$_{liquid}$=60% L (times are presented in hours:minutes since the start of the experiment)

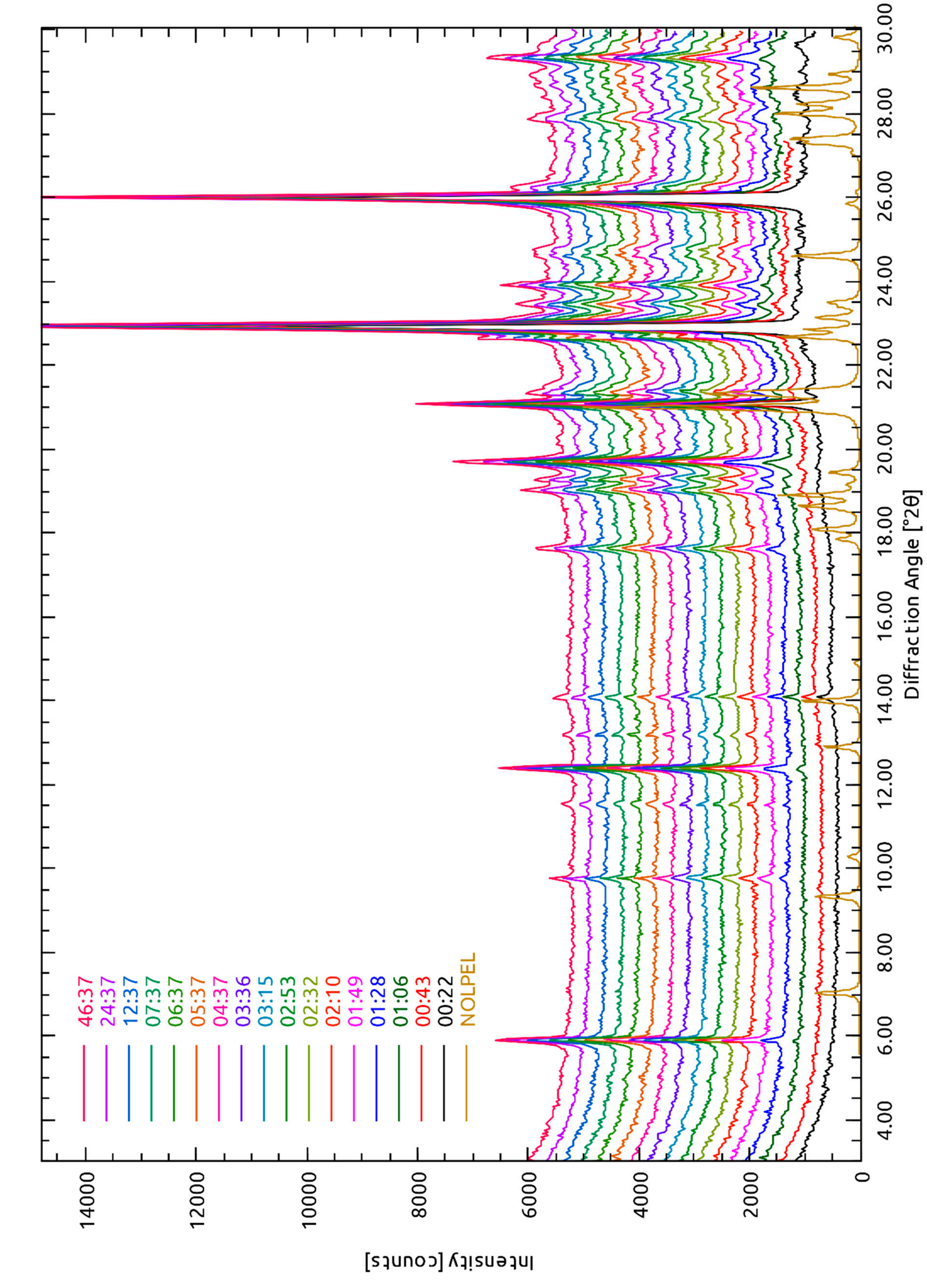

Figure S3: In-SituX XRPD patterns, e.e.$_{solid}$=1.0% D e.e.$_{liquid}$=71% L (times are presented in hours:minutes since the start of the experiment)

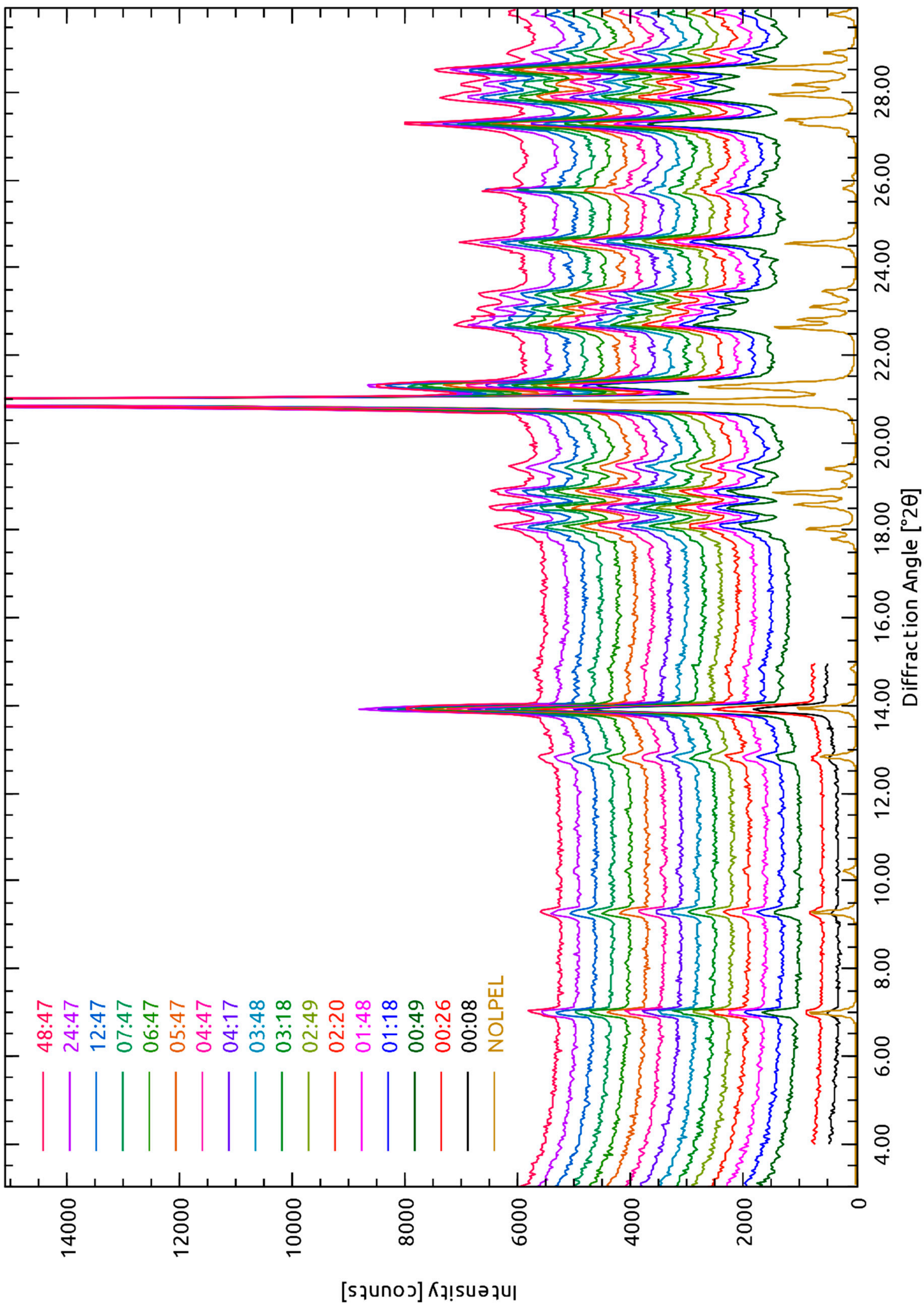

Figure S4: In-SituX XRPD patterns, e.e.$_{solid}$=2.0%D, e.e.$_{liquid}$=46% L (times are presented in hours:minutes since the start of the experiment)

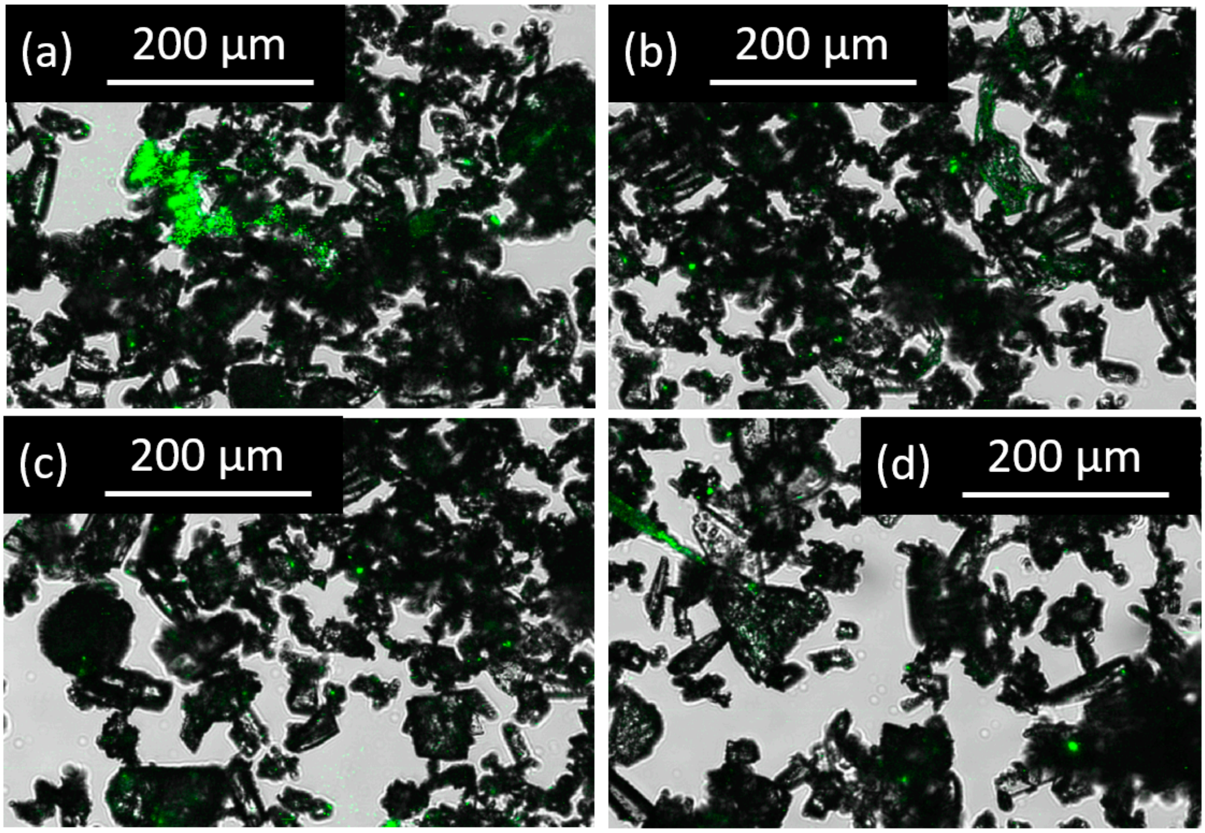

Figure S5: Enlargement of the SHG microscopy pictures of the ArgFum crystals obtained by using the standard protocol.

Table S5. Computed energy (kcal/mol) from reference structure and modeled solid solutions with 1, 2, and 3 substitutions.

Structure	Reference Structure			Solid Solution		
Number of permutations	1	2	3	1	2	3
E_{lattice}		-27163.97		-27163.14	-27151.21	-27144.18
E_{guest}	-237.12	-474.25	-711.37	-238.33	-473.22	-708.56
E_{host}	-26780.16	-26400.62	-26025.35	-26774.77	-26391.39	-26003.91
U_{lattice}	-146.69	-289.11	-427.25	-150.04	-286.59	-431.71
ΔU		N/A		**-3.35**	**2.51**	**-4.46**

Figure S6. Representation of structures of Arginine fumarate (left), and structure obtained after enantiomeric substitution.

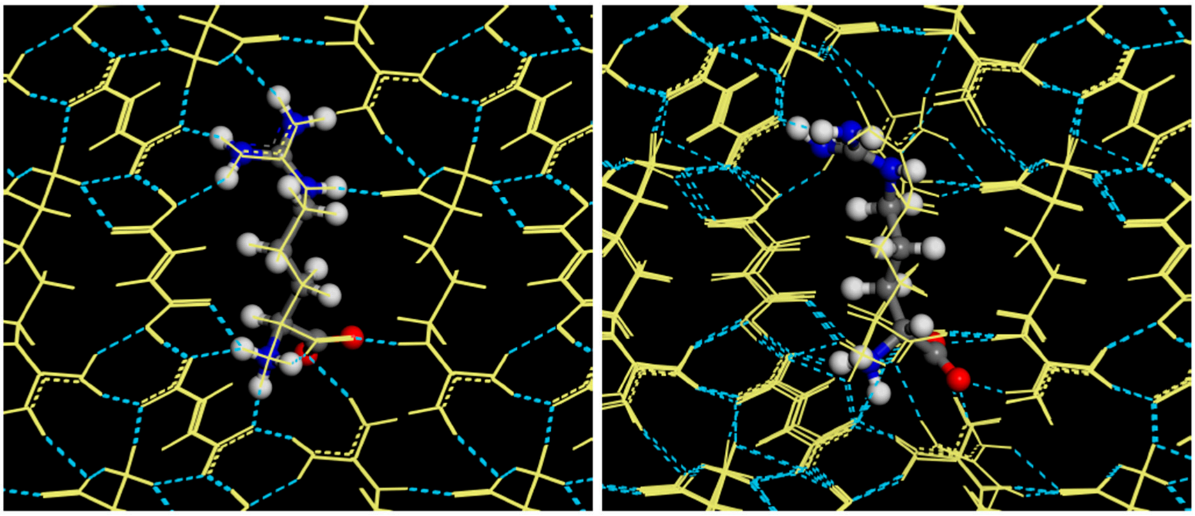

Supplement: Supplementary file 1 [file molecules-27-08652-s001.zip › molecules-2021921-supplementary materials.pdf]
